# PD_Manager: an mHealth platform for Parkinson's disease patient management

**DOI:** 10.1049/htl.2017.0007

**Published:** 2017-05-23

**Authors:** Kostas M. Tsiouris, Dimitrios Gatsios, George Rigas, Dragana Miljkovic, Barbara Koroušić Seljak, Marko Bohanec, Maria T. Arredondo, Angelo Antonini, Spyros Konitsiotis, Dimitrios D. Koutsouris, Dimitrios I. Fotiadis

**Affiliations:** 1 Biomedical Engineering Laboratory, School of Electrical and Computer Engineering, National Technical University of Athens, GR15773 Athens, Greece; 2 Unit of Medical Technology and Intelligent Information Systems, University of Ioannina, GR45110 Ioannina, Greece; 3 Department of Knowledge Technologies, Jozef Stefan Institute, Jamova 39, SI1000 Ljubljana, Slovenia; 4 Computer Systems Department, Jozef Stefan Institute, Jamova 39, SI1000 Ljubljana, Slovenia; 5 Life Supporting Technologies, Universidad Politécnica de Madrid, Avenida Complutense 30, ES28040 Madrid, Spain; 6 Department for Parkinson's Disease, IRCCS San Camillo, Via Alberoni 70, IT30126 Venice, Italy; 7 Department of Neurology, Medical School, University of Ioannina, GR45110 Ioannina, Greece

**Keywords:** diseases, mobile computing, telemedicine, patient monitoring, smart phones, cognition, decision support systems, accelerometers, gyroscopes, optical sensors, bio-optics, cardiology, temperature sensors, skin temperature sensor, optical heart rate sensor, gyroscope, accelerometers, DSS, decision support system, cloud infrastructure, nutrition, mood, cognition, nonmotor self-evaluation tests, PD motor symptoms, smartphone, insole sensors, wrist sensors, Parkinson's disease patient management, mobile health platform, mHealth platform, PD_Manager

## Abstract

PD_Manager is a mobile health platform designed to cover most of the aspects regarding the management of Parkinson's disease (PD) in a holistic approach. Patients are unobtrusively monitored using commercial wrist and insole sensors paired with a smartphone, to automatically estimate the severity of most of the PD motor symptoms. Besides motor symptoms monitoring, the patient's mobile application also provides various non-motor self-evaluation tests for assessing cognition, mood and nutrition to motivate them in becoming more active in managing their disease. All data from the mobile application and the sensors is transferred to a cloud infrastructure to allow easy access for clinicians and further processing. Clinicians can access this information using a separate mobile application that is specifically designed for their respective needs to provide faster and more accurate assessment of PD symptoms that facilitate patient evaluation. Machine learning techniques are used to estimate symptoms and disease progression trends to further enhance the provided information. The platform is also complemented with a decision support system (DSS) that notifies clinicians for the detection of new symptoms or the worsening of existing ones. As patient's symptoms are progressing, the DSS can also provide specific suggestions regarding appropriate medication changes.

## 1 Introduction

Parkinson's disease (PD) is a complex chronic disease that most people live with for many years or even decades. It is the second most common neurodegenerative disease and its prevalence will continue to grow as the population ages [1]. Since medical understanding regarding neurodegenerative diseases, such as PD, is still in a primitive research status and far from providing a cure, better disease management and patient monitoring need to fill the gap and ensure that the patient retains his independence and continues to have the best quality of life possible. Due to its complexity, achieving an optimal management of PD is a rather difficult task that requires a multidisciplinary approach, involving the collaboration of different types of medical experts including neurologists, physiotherapists, psychologists and dieticians. On the other hand, patient's quality of life dictates new rules and standards in monitoring devices, highlighting the need for accurate and continuous monitoring with minimum intervention to everyday life activities, comfort and ease of use. Considering these requirements, PD_Manager mHealth platform offers a holistic mHealth ecosystem, based on the principles of internet of things, to address every aspect regarding better management of PD patients.

From the patient's perspective, the aim is to use only a few lightweight sensors that can unobtrusively evaluate all the primary PD symptoms with minimum effect on daily activities. The patient's main requirement is to follow the clinician's instructions that are obtained using a specifically designed mobile application (e.g. medication plan, nutrition diet and perform cognitive/motor self-evaluation tests). Furthermore, informative material regarding PD management and best practice examples are also provided to facilitate everyday life and educate patients, caregivers or even non-expert healthcare providers. In this way, patients become active co-actors in the management of their disease, gaining better and more personalised treatment.

From the clinician's perspective, the mHealth platform offers a unified system for accurate patient monitoring and PD symptoms assessment, by enhancing the way information about patient's current condition and disease progression are provided, which enables faster evaluation. Furthermore, the amount of visits required is drastically reduced since symptom evaluation can be remotely performed in patient's home environment. All gathered data is transferred to a central cloud infrastructure and, thus, are always available and can be simultaneously accessed by different clinicians who can suggest appropriate actions regarding their field of expertise, and inspect the patient's response. In this way, the mHealth platform cannot only monitor patients but also evaluate different PD management plans and provide useful information regarding their success in controlling PD symptoms, psychological complication, atypical disease progression rates, patients' compliance etc.

Similar commercial and research systems targeting PD management are also available. The Parkinson's KinetiGraph Data Logger [2] is a wrist worn medical device that the patient can wear to evaluate symptoms such as dyskinesia and bradykinesia. The data can be then reviewed offline by the clinician. In the same context, Kinesia 360 uses wearable sensors and a mobile app to continuously monitor PD symptoms during the day measuring tremor, dyskinesia and mobility. Kinesia ONE uses a similar app and wireless sensors to measure tremor, bradykinesia and dyskinesia during specific tasks for better accuracy [3]. There are also available mobile apps that the patients can use to help them track and report their symptoms and adherence to treatment to their clinicians for better communication [4, 5] and initiatives that promote public PD data gathering [6]. In a research approach PERFORM, REMPARK and SENSE-PARK [7–9] introduced novel closed-loop systems that make use of various wearable sensors (mainly accelerometers and gyroscopes) to monitor and evaluate motor symptoms and allow the clinicians to better formulate their treatment.

Following technological advances, PD_Manager goes beyond the state of the art by combining a mHealth approach in a cloud-based infrastructure, with real-time and continuous patient monitoring using a smartphone and only three lightweight and unobtrusive sensors, namely a wristband and an insole sensor for each foot. Data capturing with the pressure sensors of the insole sensors in particular has never been used for PD symptoms evaluation in the past. Furthermore, PD_Manager follows a holistic approach regarding the management of the disease, covering almost every aspect affecting patient's condition and progression, while enhancing data and knowledge communication between the users of the ecosystem (i.e. patients, caregivers and healthcare providers). This holistic approach is unique to PD_Manager even when compared with similar commercially available applications. Finally, the integrated decision support system (DSS) has been developed to facilitate patient monitoring and treatment planning. It is designed to automatically capture PD-related changes in patient's symptoms and behaviour to inform and/or alert clinicians about disease progression and suggest appropriate actions based on machine learning techniques or medical knowledge.

## 2 mHealth system architecture

**Fig. 1:**
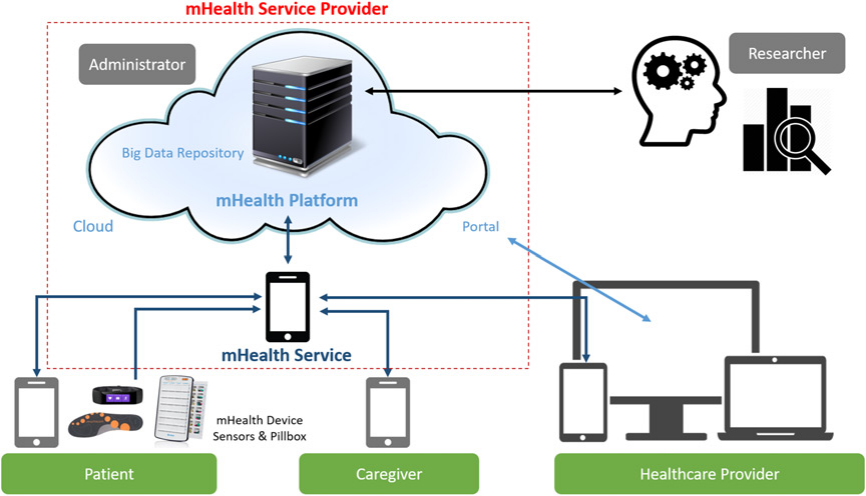
Schematic representation of PD_Manager's mHealth platform cloud-based architecture

Although the PD_Manager platform represents an entity, the system can be decomposed into some primary components as shown in Fig. 1. The platform connects patients, healthcare providers, caregivers, researchers and commercial IT solutions via an extendable architecture designed to maximise modularity and scalability by easily adding, upgrading and changing components. The core of the system is the mHealth platform which is a cloud IT system that provides all the necessary functionality for users and services communication, along with compute power for data processing and storage. This platform is supported by the mHealth Service Provider. It is built upon 3DNet [10], which is a commercial cloud technology system developed to establish common accepted communication standards in healthcare industry.

The mHealth Service is responsible for establishing communication and data transfer between the cloud platform and the mobile applications over the internet. The service ensures that the data from the sensors is successfully transferred following security and data encryption protocols from the patient's smartphone to the cloud. All devices and sensors that are used for patient monitoring are termed as mHealth devices and must use the mHealth service to successfully connect to the platform, without requiring any intervention from the patient. The primary users and roles are also depicted in Fig. 1 and include (i) the patient suffering from PD, (ii) the patient's caregiver assisting the patient in everyday tasks, (iii) the healthcare provider medical experts (e.g. neurologists, nurses, physiotherapists, nutritionists, psychologists etc.), (iv) the researcher who performs post analysis to extract data-oriented knowledge and provides clinical decision support solutions to assist healthcare providers and (v) the administration who is responsible for system maintenance, technical support and user/roles management.

## 3 Devices and sensors

### 3.1 Wrist sensor

In a very rapidly changing scene where newer devices are regularly released, the Microsoft Band was selected as it offered the wider range of sensors for patient monitoring in a very compact and lightweight device [11]. It includes three-axis accelerometer/gyroscope sensors, optical heart rate sensor, skin temperature sensor, capacitive and galvanic skin response sensors and an ultraviolet sensor. In addition, a very useful feature is the integrated display which can be used to provide notifications and alerts to the patient. Another advantage of the Microsoft Band is its Software Development Kit (SDK) which is free to download and, despite being recently discontinued, has a large community of independent developers supporting each other. Through SDK developers gain direct access to sensors and raw data, as well as the ability to develop application-specific functionality; features that are essential to cover project's specifications. The band is also supported by Microsoft Health, a software that provides useful insights regarding sleep quality and patient's activity level.

### 3.2 Sensor insoles

The sensor insole is an innovative device developed and produced by Moticon [12]. It has a shoe sole design being flexible, flat and lightweight, providing easy setup and comfortable use. The sensor insole provides a great solution to unobtrusively measure patient's foot pressure distribution, acceleration pace, weight-bearing, balance, stability and motion sequences. To perform the above measurement, the sole contains 13 capacitive pressure sensors that are scattered across the entire foot area, an embedded 3D acceleration sensor and a temperature sensor. The raw sensor data is directly processed in the embedded microcontroller which computes a variety of essential gait and motion parameters. Then, these parameters can either be streamed in real-time for live view or stored in the on-board memory for future download. The software that accompanies the sensor insole simplifies the evaluation of patient's walking behaviour. It allows completely mobile gait and motion analysis providing reports on gait and walking characteristics such as gait lines, mean pressure distribution, temporal gait parameters, contact forces, leg extension power, lack of balance and lack of symmetry between the left and right leg.

### 3.3 Pillbox

The use of a smart pillbox is proposed to optimise medication intake, since following the medication plan carefully is crucial for effective control of PD symptoms. The pillbox can be filled in advance by pharmacists or caregivers according to the prescribed medication plan and then handed over to the patients for intake. Based on project specifications, the SimpleMed+ pillbox from Vaica [13] was selected for monitoring medication intake, since it was the only commercial solution that provided an SDK within a reasonable cost range. SimpleMed+ has its own GSM connectivity cloud services that can be easily integrated through an open application programming interface to the mHealth platform. It can store solid medication in separate compartments for an entire week with dosage separation into four intervals. The schedule is automatically downloaded from the cloud service and the patient is alerted accordingly. The pillbox alerts the patient when it is time to take a medicine. If a dosage is missed, notifications are sent to caregivers and healthcare providers.

### 3.4. Smartphone

Besides the cloud service, most of the mHealth functionality is carried through the patient's mobile application (i.e. data transfer, motor/non-motor tests, nutrition, educational material etc.). This applies for clinicians as well, who can access all data and perform patient evaluation through the corresponding medical mobile application. The most popular mobile operating systems (i.e. Android, iOS and Windows phone) are supported to promote the use of existing infrastructure and avoid adding extra hardware costs to patients, caregivers and healthcare providers due to compatibility issues.

## 4 Patient evaluation parameters

An initial set of clinical parameters is collected upon baseline registration from the clinician. Besides demographic information, these baseline parameters include patient's age, initial diagnosis, side of onset, disease duration, current symptoms, previous and current treatment (duration and dosages in Levodopa equivalent daily dosage and dopamine agonist equivalent daily dosage), years under levodopa, presence or history of psychiatric disease, psychiatric treatment and/or other comorbidities. The majority of the PD symptoms evaluation parameters, however, are automatically extracted from the sensors described above and the tests available through the mobile application. The evaluation includes estimation of motor symptoms, cognition, speech, quality of life and sleep and level of daily activity.

Depending on the studied PD symptom the most appropriate sensor data and devices are utilised, while some symptoms are estimated from a combination of sensors and/or mobile apps. Moticon's sensor insole is used to evaluate PD symptoms related to gait and balance based on readings from the embedded pressure and accelerometer sensors. Microsoft Band is primarily used to evaluate PD symptoms expressed at the upper extremities such as hand tremor (both rest and postural) and rigidity utilising the accelerometer and gyrometer sensors. The complete list of all the symptoms that are supported and can be evaluated is presented in Table 1, along with the corresponding device and type of sensor.

**Table 1:** Symptoms evaluated using sensors and mobile devices

PD symptom	Sensor used	Device
tremor at rest	accelerometer, gyroscope	wristband
postural tremor	accelerometer, gyroscope	wristband
dyskinesia intensity	accelerometers, gyroscopes	wristband
dyskinesia Duration	pressure, accelerometers, gyroscopes	smartphone, wristband, insoles
bradykinesia	pressure, accelerometers, gyroscopes	smartphone, wristband, insoles
gait	pressure, accelerometers	wristband, senor insole
FoG	pressure, accelerometers	senor insole
OFFs duration	pressure, accelerometers, gyroscopes	smartphone, wristband, insoles
finger tapping	—	smartphone
cognition	—	smartphone
reading ability	microphone	smartphone
mood	microphone	smartphone
logical inferences	microphone	smartphone
sleep (restful)	accelerometers, gyroscopes	wristband
sleep (light)	accelerometers, gyroscopes	wristband
activity distance	accelerometers, gyroscopes	wristband
steps	pressure, accelerometers	senor insole
activity time	accelerometers, gyroscopes	wristband
medication intakes	—	pillbox

## 5 mHealth system functionality

### 5.1 Mobile app for patients

**Fig. 2:**
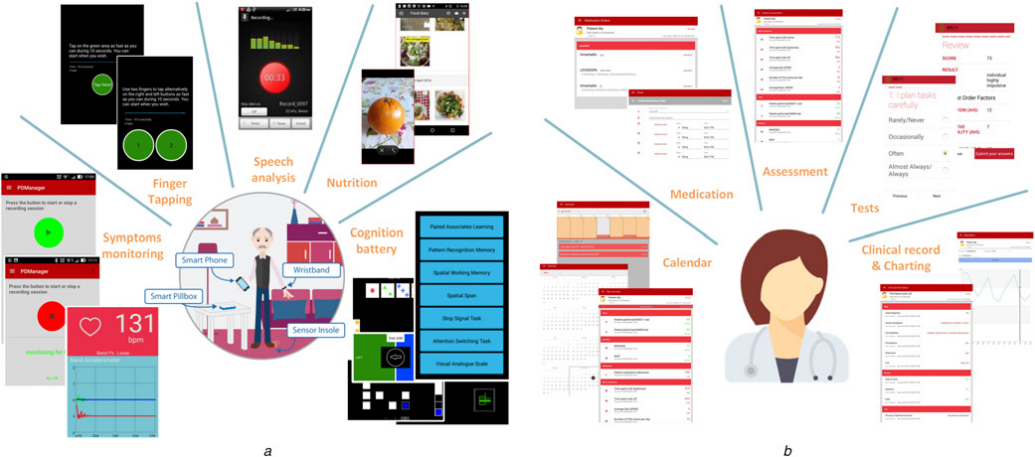
Mobile application functionality for *a* Patients *b* Healthcare providers

The Patient app is used to implement the majority of the tests for motor, cognitive, speech, mood and nutrition assessment. The design of the interface was implemented targeting elderly people with mild or severe motor impairments. Every part of the user interface contains clear instructions in text, which are also reproducible phonetically. The primary functionality modules available for patients are shown in Fig. 2*a* and presented in the following sections.

#### 5.1.1 Sensors monitoring module

This component is used to enable patient monitoring with the sensors. The recording begins by tapping a green play button on the screen and terminated from a similar red stop button. If an issue emerges the app alerts the patient and/or caregiver and proposes a possible solution. Some sensor data can be shown in real time if necessary. A very useful feature is the ability to alert the patient when the battery of the sensors is running low to recharge the devices. The monitoring system was designed to require the minimum user intervention possible (i.e. start/stop the recording session and recharge the batteries).

#### 5.1.2 Finger tapping module

This module is also part of motor symptoms evaluation and consists of two tasks. In the first task, a green circle is displayed on the screen. The participant is instructed to tap with one finger inside the designated area as fast as possible for 10 s. A 10 s counter begins when the patient makes the first tap. When this test is completed the second task, containing the alternate finger tapping test, is automatically loaded. In this test, there are two green areas displayed on the screen. The participant is instructed to use two fingers and tap alternatively on the left and right circle as fast as possible for the same 10-s duration.

#### 5.1.3 Cognition battery module

The evaluation of cognitive impairment is performed using an adaptation of the widely used Cambridge Neuropsychological Test Automated Battery that has been extensively validated for elderly populations and PD patients [14]. Seven tests are available in the patient app including Paired Associates Learning Test that assesses visual memory and new learning skill, Pattern Recognition Memory Test that assesses visual pattern recognition memory, Spatial Working Memory Test that assesses retention and manipulation of visuospatial information, Spatial Span Test that assesses working memory capacity, Stop Signal Task Test that assesses response inhibition (impulse control), Attention Switching Task Test that assesses the ability to focus and Visual Analogue Scales Test that assesses subjective states of drug effect, energy levels, sickness, alertness and mood. The main advantages of these tests are that they are language and country independent, they do not require a test administrator and were specifically designed for being presented in a touch screen.

#### 5.1.4 Speech analysis module

This component evaluates speech-related parameters based on features extracted from patient's voice. There are two tests available. The first is speech analysis which evaluates cognitive and mood aspects by analysing continuous speech. Speaking rate parameters such as number of pauses, length of pauses, length of phrases, duration of spoken syllables, voice onset times and sentence duration are extracted through a reading test [15]. Patient's mood is estimated based on positive/negative polarity of words and the above extracted parameters from the spoken content. Logical inference is estimated through a logical test in speech modality. The second is the sound analysis test where patients are instructed to perform a sustained phonation sound (e.g. ‘AAAH’) for 3–5 s to automatically assess motor aspects of PD patient's speech. For better recording performance, the patient is instructed to place the smartphone in close proximity.

#### 5.1.5 Nutrition module

Nutrition status of PD patients is often not taken into consideration until evident signs of body mass loss are noticeable. To tackle this issue and motivate patients in adopting healthy eating habits, they are promoted in collecting data about their everyday diet using this module. This is accomplished by taking photos of what they are about to eat using the smartphone and tagging the photos with food names and portion sizes. Since photo tagging might be a difficult or tedious task for elders and especially PD patients, an image recognition algorithm is developed to automatically classify images with respect to food and dish categories [16]. Nutrition monitoring is built around the Open Platform for Clinical Nutrition, OPEN (http://opkp.si), which is an open access, web-based application that supports diet planning and automatic food recognition. Synchronisation between OPEN and the patient's smartphone is automatically performed if a connection with the internet is available.

### 5.2 Mobile app for healthcare providers

For healthcare providers and clinicians, a separate mobile application has also been developed and adjusted to their specialised needs. The application's design focused on providing fast patient assessment, considering the busy schedule of clinicians in every day practice. The primary modules of the Clinician's app are shown in Fig. 2*b* and presented below.

#### 5.2.1 Clinical record module

The most relevant features regarding the patient's condition can be easily assessed from this brief patient record tab. The majority of the clinical parameters collected during patient registration, as mentioned in Section 4, and the most important current PD motor symptoms are presented here. This module can be regarded as a very compact electronic record for each patient, offering easy access to all history data from anywhere. To provide a more detailed representation of patient data chart modalities are supported within the Clinician's app.

#### 5.2.2 Patient assessment module

This module consists the basic tool to allow fast and comprehensive presentation of all the PD parameters that are extracted during monitoring with the various sensors. It is the easiest way to inspect PD symptoms worsening trends and disease progression overall. It provides information regarding patient's motor symptoms evaluation such as gait and bradykinesia and symptoms trends such as time spent under tremor, dyskinesia and in OFF state as percentage during a day. Values that represent severe symptoms for any of the above parameters are highlighted in red colour to declare issues that might require clinician's attention. Results from other non-motor tests performed by the patient remotely using the patient's app or by other clinicians in previous visits (including sleep analysis and nutrition) are also presented to provide a complete assessment of patient's condition.

#### 5.2.3 Calendar module

The calendar is very useful tool for faster patient evaluation over prolonged periods of time (e.g. from one visit to another). The app offers powerful customisation options and various features. The dates in the calendar can be presented with different modes extending from a single week to an entire year. The most useful feature is that the calendar offers event integration per day. In this context, alarming behaviour that was noted at some period during 1 day and is related to PD symptoms (motor and/or non-motor) is shown in red coloured font and can be easily indexed.

#### 5.2.4 Digital evaluation tests module

The clinician's app includes a framework that allows questionnaires and assessment scales that are used during patient visits to be easily integrated. Currently, the digitised versions of the non-motor symptoms scale [17] and the Barratt impulsiveness scale (BIS-11) [18] are available for the clinicians. Similar tests that require professional administration to be appropriately conducted and, thus, cannot be included in the Patient's app can be implemented here. These type of tests have to be performed during patient visits.

#### 5.2.5 Medication module

This component allows the healthcare provider to review the current and past medication plans and register a new one if required. The review tab provides information about the name of the medicine and the date and time of the last five intakes as acquired from the pillbox. Full history of intakes is available upon selecting a particular medicine. The current plan can be modified by deleting existing records or adding new ones. In the latter case, the prescribing clinician must specify the type of medicine, the number of intakes per day, the time of first intake and the suggested dosage per intake. This information is then transferred to the pillbox to update the medication schedule. Notifications for medication changes are also sent to patients and caregivers.

#### 5.2.6 Nutrition module

All data collected by the patient's nutrition app are transferred in OPEN, where detailed analysis of food diary is performed and data is prepared for usage by nutrition experts. The clinician's nutrition module is more complex and supports the collection of patient's personal and anthropometric data (weight, height etc.), results of bioelectrical impedance and biomarkers data analysis and information about their medication. It also integrates a malnutrition screening tool (NRS-2002), a dysphagia screening tool (MDS) and a food frequency questionnaire. The majority of the above functionality is supported by OPEN and the results are then presented to the clinician through the mobile app.

### 5.3 Notifications and alerts system

This system is responsible for creating, sending and receiving notifications in different formats including SMS, e-mails and Google Play notifications. Alerts and notifications can be created from sensors, users and other systems, such as the DSS. Each alert has a corresponding user or group of users that should be forwarded to. This module receives alerts and depending on its settings decides, where, when and how to send each notification. Automated alerts can be generated and sent if patient's parameters exceed certain thresholds that declare a condition that might require immediate attention (e.g. medication changes to better control PD symptoms). Notification and reminders can also be automatically dispatched to ensure the best compliance with the PD management plan (e.g. notify patients for an incoming intake and alert caregiver if missed). Manual notifications can also be created when a user needs to interact with another to perform a specific task. For example, if required the clinician can instruct the patient to perform a specific self-evaluation test from the smartphone or start a monitoring session using the sensors.

### 5.4 Educational gallery

PD patients and untrained caregivers (e.g. close relatives) are sometimes lucking basic information regarding symptoms expression, disease progression and ways to better deal with PD-related difficulties in everyday life. A list of 11 videos with professional actors and production team are available, reflecting some of the most common difficulties of a patient suffering from PD. They focus on providing useful tips and solutions to show to both patients and caregivers ways to overcome common disease-related problems (e.g. depression, hand writing, swallowing, falls etc.) and retain the best quality of life possible.

## 6 Results

Since motor symptoms evaluation is the most important aspect of PD management, the motor symptoms monitoring module has been tested in a pilot study with 20 patients. A medical protocol was defined to assess patients' gait, freezing of gait events, tremor, dyskinesia and bradykinesia from the sensors. The assessment values that the medical experts assigned in the corresponding MDS-UPDRS items [19] were used to validate symptom classification performance.

Initially, a subset of 17 patients was usable for estimating gait problems. For each patient eight sessions in which patients performed a set of everyday activities following the protocol were recorded. The results showed that in 69% of the cases the gait score was correctly estimated in accordance to the clinician's MDS-UPDRS gait annotation, while only 3% of the estimations deviated by more than one value from the clinician's assessment. In addition, the accuracy for detecting the presence of gait impairments (i.e. MDS-UPDRS gait value > 0) reached 91%. For the detection of freezing of gait (FoG) events, only the sessions of seven patients could be used, since the rest of the patients did not express any FoG event. In total, 45 FoG events with an average duration of 33 s were marked in all sessions and 91% of them were successfully detected and classified according to the corresponding MDS-UPDRS FoG value.

Regarding tremor detection, the platform is able to estimate both amplitude and constancy for rest and postural tremor. Data from 11 patients performing the protocol of everyday activities while being monitored was used for tremor evaluation. In total, 50 sessions were available consisting of 1567 segments with tremor and 4651 segments with no tremor present. Segments had a 3-s window duration. The presence of tremor could be detected with 94% accuracy, while amplitude estimation had an 88% accuracy across all classes [20]. The clinicians' scores for the corresponding MDS-UPDRS tremor items were used to validate the estimated tremor amplitude values as before.

For the evaluation of dyskinesia detection, the dataset consisted of 38 sessions with an average duration of 20 min. For a signal processing window of 5–30 min, the detection accuracy was between 88 and 98%. The classification results for the discrimination of dyskinesia intensity between slight–mild group and moderate–severe group presented 92% accuracy. Finally, to evaluate the detection of patients' bradykinesia, 13 patients were monitored while performing different everyday life activities in ON and OFF states (i.e. sitting, standing, walking, lying down and drinking water). Based on these measures, the system estimated each patient's bradykinesia value that was then validated with the clinician's value assigned for the corresponding MDS-UPDRS item. The classification accuracy reached 82% using a 30-s window for signal processing.

The patients were also asked if they could use this type of mHealth platform to better manage their disease. From the 18 participants that filled a small questionnaire 14 (or 78%) responded that they would use it, with the main reason being their feeling of assurance due to close and more personalised treatment.

## 7 Decision support system

The mHealth system of PD_Manager is accompanied by a build-in DSS system. The first part of the DSS is a disease progression and symptoms worsening monitoring that alerts clinicians if new PD symptoms are detected (e.g. a patient with early signs of gait impairment) or critical changes in existing ones have occurred (e.g. increased OFFs duration). The second part is the evaluation of patient's adherence to the medication plan by evaluating data gathered through the pillbox. The different adherence measures are weighted to provide an overall adherence measure for each patient. The patient is then compared with similar adherence patient groups to define the severity of his adherence from very common incidents of lower significance (e.g. missing one dosage per month) to rare that require immediate attention since otherwise severe implications are to be expected. The third part of the DSS provides insights for medication modifications, by suggesting that a potential change should be considered and also the appropriate change in the current medication (i.e. adding a new substance or increasing the dosage of the already prescribed drug). This is performed by combining medication plan simulation scenarios with medical knowledge from experienced clinicians and international guidelines using the qualitative multi-criteria method Decision EXpert [21]. The DSS takes into consideration all the possible combinations of the available PD drugs (i.e. MAO inhibitors, dopamine agonists and levodopa) and proposes the optimal solution depending on which PD symptoms have worsen and if any drug or dosage limitations apply. All the above DSS functionality is developed using the collected data (e.g. the pilot study), as well as open PD patient data such as Parkinson's Progression Markers Initiative (www.ppmiinfo.org/data).

## 8 Security

This section presents the protocols applied in the PD_Manager mHealth platform regarding data security. The platform has been developed following the Fast Healthcare Interoperability Resources (FHIR) framework, which is the next generation of HL7 standards, that was created targeting communication in large institutional healthcare providers. FHIR is not a security protocol itself, but defines exchange protocols and content models that need to be used to enhance system's security. Regarding communication security, all data is transferred using hypertext transfer protocol, HTTP, within a connection encrypted by transport layer security or secure sockets layer (TLS/SSL). The TLS/SSL encryption is performed prior to any HTTP communication, so the whole interaction is protected. The security of the endpoints is also risk-managed by storing audit logs that are generated by every module of the platform, allowing usage tracking of modules and users. The client/users authentication interfaces are implemented following the OAuth 2.0 authorisation framework specifications, which is an open protocol that enables secure authorisation for web based, desktop and mobile applications. Finally, access control management is implemented using a user authorisation system that is responsible to define the content that each user is allowed to access and manage.

## 9 Conclusion

The PD_Manager mHealth platform has been designed and developed to provide a holistic solution that tackles most aspects regarding PD patients management. The aim is to motivate patients and caregivers in becoming more active and efficient in their treatment and facilitate clinicians in everyday practice by providing tools that enable faster and more accurate evaluation of patients' condition. The platform promotes the collaboration between medical personnel with different fields of expertise as well as between clinicians and patients/caregivers to improve PD management plans and eventually patients' quality of life.
